# High‐Density Spectral Analysis for Bi‐Atrial Arrhythmogenic Substrate During Non‐Paroxysmal Atrial Fibrillation

**DOI:** 10.1002/joa3.70477

**Published:** 2026-09-21

**Authors:** Nobunari Tomura, Keitaro Senoo, Taku Ohkawa, Yusuke Kakei, Kohei Kawai, Masahiro Makino, Jun Munakata, Tomonori Miki, Yukinori Kato, Kento Fukui, Hiroki Kitajima, Suguru Nishiuchi, Ken Kakita, Daisuke Sato, Hirokazu Shiraishi, Satoaki Matoba

**Affiliations:** ^1^ Department of Cardiovascular Medicine, Graduate School of Medical Sciences Kyoto Prefectural University of Medicine Kyoto Japan; ^2^ Department of Cardiac Arrhythmia Research and Innovation, Graduate School of Medical Sciences Kyoto Prefectural University of Medicine Kyoto Japan; ^3^ Department of Cardiovascular Medicine Kyoto Tanabe Central Hospital Kyoto Japan; ^4^ Department of Cardiovascular Medicine Kyoto Chubu Medical Center Kyoto Japan; ^5^ Department of Cardiovascular Medicine Kyoto Okamoto Memorial Hospital Kyoto Japan; ^6^ Beppu Medical Clinic Kyoto Japan; ^7^ Department of Cardiovascular Medicine Gunma Prefectural Cardiovascular Center Maebashi Japan; ^8^ Department of Cardiovascular Medicine Koseikai Takeda Hospital Kyoto Japan; ^9^ Department of Cardiovascular Medicine Tenyokai Central Hospital Kagoshima Japan

**Keywords:** atrial fibrillation, CARTOFINDER, dominant frequency deviation, regularity index, spectral analysis

## Abstract

**Background:**

Spectral analysis has been utilized to identify localized sites exhibiting high dominant frequency (DF) and high regularity—the critical arrhythmogenic substrate driving atrial fibrillation (AF). This study aimed to elucidate the spectral characteristics of focal activation (FoA) and rotational activation (RoA) sites during AF using a high‐density mapping system.

**Methods:**

CARTOFINDER mapping was prospectively performed in both atria of 47 consecutive non‐paroxysmal AF patients using the Octaray catheter prior to ablation. Endocardial unipolar electrograms were exported offline for spectral analysis of DF and the regularity index (RI).

**Results:**

CARTOFINDER assessed a total of 48 153 mapping points. Given substantial inter‐subject variability in mean DF, the difference between each point's DF and the subject‐level mean DF was defined as the dominant frequency deviation (DFD). The left atrium (LA) demonstrated significantly higher DFD and RI than the right atrium (both *p* < 0.001), with the LA roof recording the highest values across all segments. Both FoA and RoA sites exhibited significantly higher DFD and RI compared with sites without FoA and RoA (both *p* < 0.001). DFD and RI increased progressively with increasing FoA repetitions (both *p* < 0.001). In contrast, high‐repetition RoA sites (> 16 repetitions) showed a significantly higher RI (0.42 ± 0.13 vs. 0.36 ± 0.12, *p* < 0.001) but a paradoxically lower DFD (0.14 ± 0.64 Hz vs. 0.26 ± 0.74 Hz, *p* < 0.001) compared with low‐repetition RoA sites.

**Conclusions:**

The combination of CARTOFINDER mapping and spectral analysis may contribute to the mechanistic understanding of non‐paroxysmal AF.

## Graphical abstract

Analyzing 48 153 CARTOFINDER mapping points revealed that both FoA and RoA sites demonstrated elevated DF deviation (DFD) and RI compared to non‐sites. While both metrics increased progressively with FoA repetitions, high‐repetition RoA sites paradoxically displayed lower values. The combination of CARTOFINDER and spectral analysis may contribute to the mechanistic understanding of non‐paroxysmal AF.

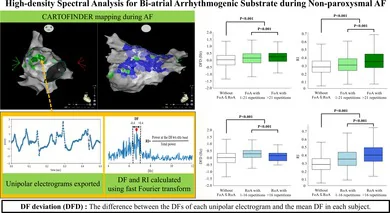

**Abbreviations**
AF: atrial fibrillation
DF: dominant frequency
DFD: dominant frequency deviation
FoA: focal activation
LA: left atrium
PV: pulmonary vein
RA: right atrium
RI: regularity index
RoA: rotational activation

## 1 Introduction

Atrial fibrillation (AF) is the most prevalent sustained cardiac arrhythmia and is associated with significantly increased risks of stroke, heart failure, and mortality [1, 2]. Pulmonary vein (PV) isolation has become the established cornerstone of catheter ablation for paroxysmal AF; however, its long‐term efficacy remains limited in patients with non‐paroxysmal AF (persistent and long‐standing persistent AF), in whom high recurrence rates represent a major clinical challenge [3, 4, 5]. To overcome this limitation, individualized ablation strategies targeting patient‐specific arrhythmogenic substrates (substrate‐guided ablation) are required, in addition to PV isolation.

Against this background, CARTOFINDER (Biosense Webster Inc.) has been developed as a high‐density panoramic mapping system that analyzes wavefront propagation in real time using unipolar intracardiac electrograms during AF. This system is capable of detecting and quantifying two distinct types of potential arrhythmogenic substrate: Focal activation (FoA)—defined as an excitation wavefront radiating centrifugally from a single focus—and rotational activation (RoA)—defined as a reentrant wavefront circulating around a defined anatomical region. Whereas previous studies employing CARTOFINDER used the conventional 20‐electrode, 5‐spline Pentaray catheter [6, 7], the present study introduces the next‐generation Octaray catheter (48 electrodes, 8 splines) to achieve higher‐density and higher‐resolution mapping.

From a spectral analysis perspective, local drivers actively sustaining AF have been shown in multiple studies to exhibit two characteristic features: Rapid excitation (high dominant frequency: DF) and highly organized, regular electrical conduction (high regularity index: RI) [8, 9, 10, 11]. Accordingly, if FoA and RoA sites identified by CARTOFINDER also demonstrate high DF and high RI on spectral analysis, this would provide additional mechanistic evidence supporting their potential role as important substrates involved in AF maintenance.

The present study hypothesized that FoA and RoA sites identified by high‐density CARTOFINDER mapping with the Octaray catheter would exhibit higher DF and RI than non‐substrate sites on spectral analysis. The study aimed to test this hypothesis and to characterize the distribution and clinical significance of arrhythmogenic substrates across both atria.

## 2 Methods

### 2.1 Study Population

Forty‐seven consecutive patients with non‐paroxysmal AF (persistent or long‐standing persistent AF) who underwent initial catheter ablation at 6 hospitals between April and December 2023 were prospectively enrolled. Exclusion criteria were: Prior history of catheter ablation and inability to obtain adequate mapping data. This study protocol conformed to the ethical guidelines of the Declaration of Helsinki (as revised in 2013) and was approved by the institutional ethics committee of Kyoto Prefectural University of Medicine (research project no. ERB‐C‐2609). Written informed consent was obtained from all patients.

### 2.2 CARTOFINDER Mapping Protocol

All antiarrhythmic drugs were discontinued for at least five half‐lives prior to the procedure to eliminate pharmacological confounding. All procedures were performed under deep sedation with propofol and dexmedetomidine hydrochloride. During the procedure, heparin was administered to maintain an activated clotting time of > 300 s. CARTOFINDER mapping was performed in both the right atrium (RA) and left atrium (LA) using an Octaray catheter with inter‐electrode spacing of either 3 to 3‐3 mm or 2–5‐2 mm according to the discretion of the individual operator, prior to ablation. Unipolar electrograms were continuously recorded for 30 s from each electrode. After filtering of far‐field ventricular signals, FoA and RoA occurring during AF were automatically detected together with their repetition counts.

**FIGURE 1:**
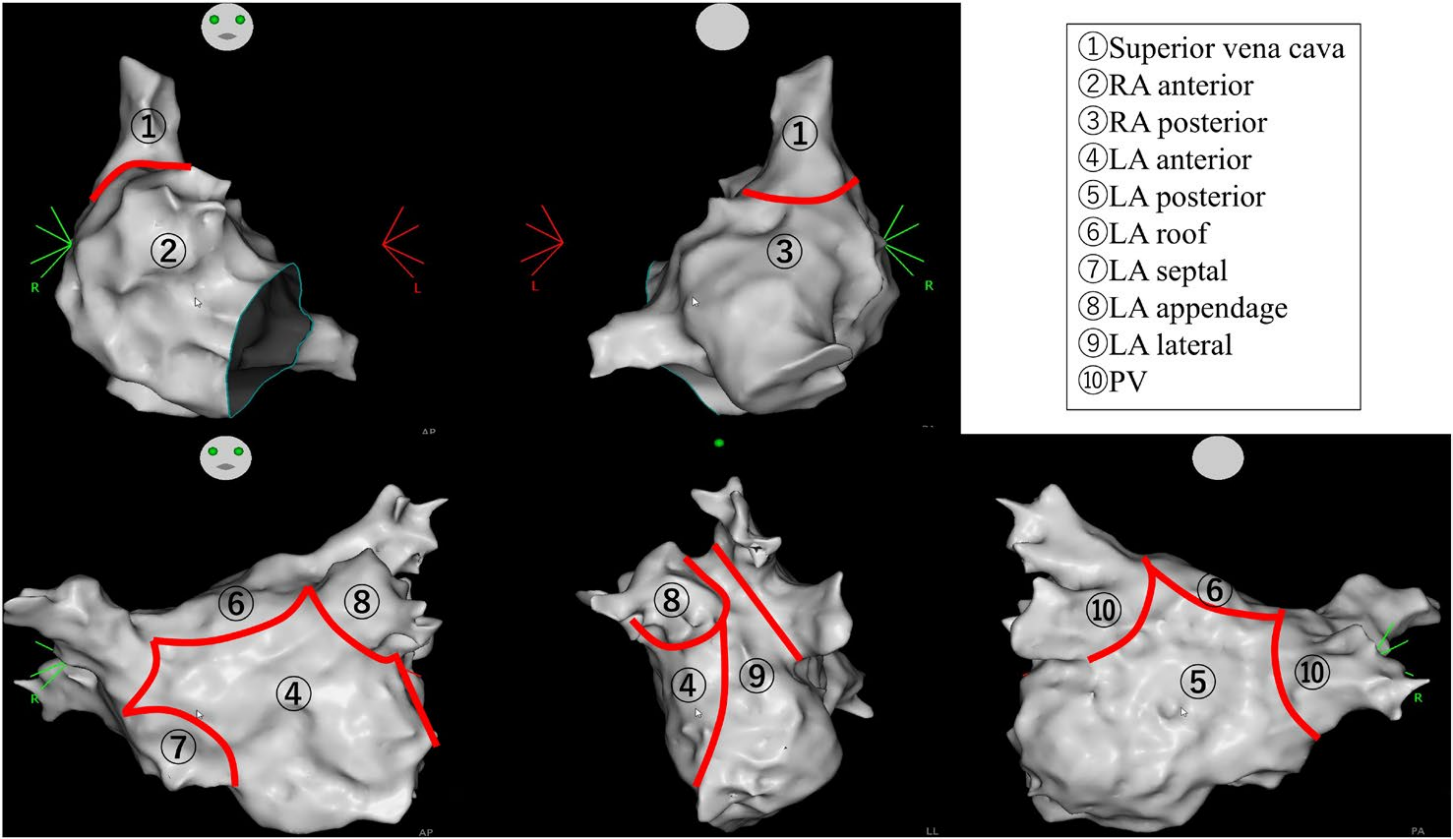
Segmented areas of both atria. Representative 3‐dimensional electro‐anatomical geometries illustrating the 10 anatomical segments used for spectral analysis. Segment boundaries are indicated by red lines. LA, left atrium; PV, pulmonary vein; RA, right atrium.

FoA was defined as the earliest activation site exhibiting a QS pattern on unipolar electrograms for ≥ 2 consecutive beats. RoA was defined as a site at which an early‐meets‐late activation sequence was observed for ≥ 2 consecutive repetitions. The mapping area was divided into a total of 10 segments: 3 in the RA (superior vena cava, anterior wall, and posterior wall) and 7 in the LA (anterior wall, posterior wall, roof, septum, LA appendage, lateral wall, and PVs) (Figure 1). Contact with the catheter was confirmed using the recorded electrograms, fluoroscopy, and 3‐dimensional geometry.

### 2.3 Spectral Analysis and Definition of Dominant Frequency Deviation

**FIGURE 2:**
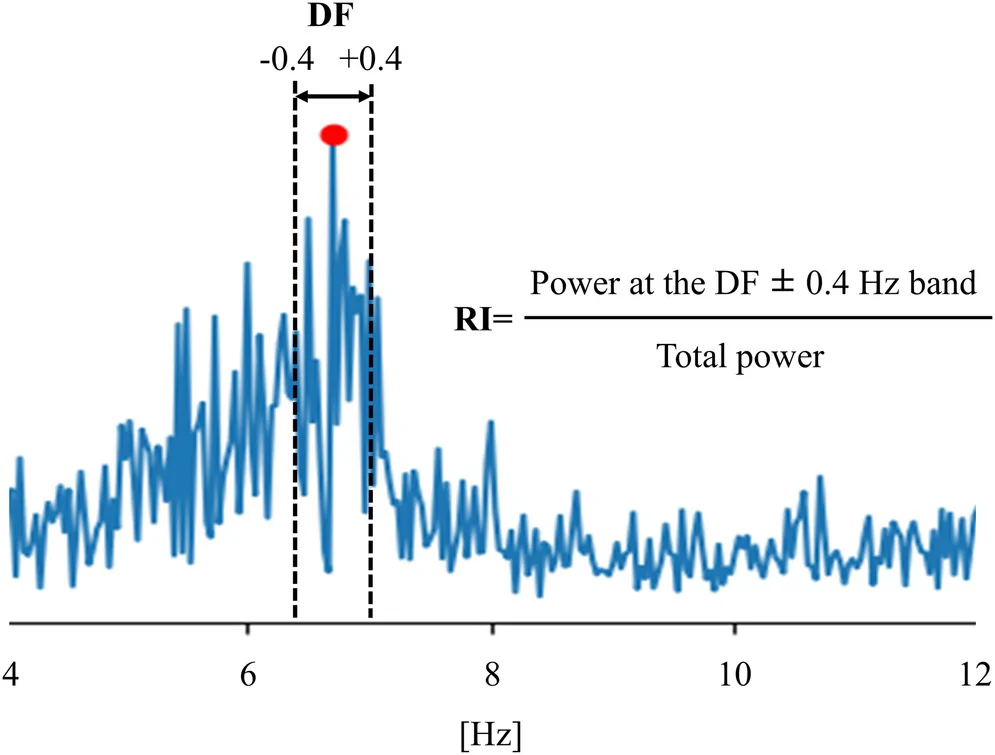
Definition of DF and RI. Representative frequency spectrum obtained by fast Fourier transform of a 30‐s unipolar electrogram (4.0–12.0 Hz). The DF (red dot) was defined as the frequency with the highest spectral power. The RI was defined as the ratio of spectral power within the DF±0.4 Hz band to total spectral power across the 4.0–12.0 Hz band. DF, dominant frequency; RI, regularity index.

Unipolar electrograms acquired by CARTOFINDER (30‐s recordings) were exported offline using dedicated software and subjected to spectral analysis by fast Fourier transform. The following two indices were calculated (Figure 2):

DF: Defined as the frequency exhibiting the highest spectral power within the 4.0–12.0 Hz frequency band [11]. DF reflects the rate (“speed”) of atrial activation at each mapping site.

RI: Defined as the ratio of spectral power within the DF±0.4 Hz band to total spectral power across the 4.0–12.0 Hz band [11]. RI reflects the regularity and organization of electrical conduction at each site, ranging from 0 to 1.Because substantial inter‐subject variability existed in mean DF (Figure S1), direct comparison of absolute DF values across patients was considered inappropriate. Therefore, the subject‐level mean DF was computed by averaging DF values across all mapping points within each patient, and this value was designated as the reference (zero baseline) for that subject. The deviation of each mapping point's DF from this reference was defined as the dominant frequency deviation (DFD) (Figure S2). A positive DFD indicates that a given site activates faster than the subject's average, whereas a negative DFD indicates slower activation, thereby enabling fair inter‐subject comparisons.

### 2.4 Statistical Analysis

Continuous variables are expressed as the mean ± standard deviation or median (interquartile range), as appropriate. Comparisons between two groups were performed using Student's *t*‐test or the Mann–Whitney U test. Categorical variables were compared using the Fisher exact test or Chi‐square test as appropriate. All statistical analyses were performed with JMP software (SAS Institute Inc.), and a *p* value of < 0.05 was considered statistically significant.

## 3 Results

### 3.1 Patient Characteristics

The baseline characteristics of the 47 patients are summarized in Table 1. The mean age was 72.0 ± 8.6 years, and 27 patients (57.5%) were male. The median AF duration was 8.4 months, with 12 patients (25.5%) classified as long‐standing persistent AF. Mean left ventricular ejection fraction was 63.2% ± 7.1%, and mean left atrial diameter was 43.8 ± 5.4 mm.

**TABLE 1:** Baseline characteristics.

	Overall (*n* = 47)
Age (years)	72.0 ± 8.6
Body mass index (kg/m^2^)	25.0 ± 3.7
Male, *n* (%)	27 (57.5)
AF duration (months)	8.4 (4.8, 12.0)
Long‐standing persistent AF, *n* (%)	12 (25.5)
Hypertension, *n* (%)	30 (63.8)
Diabetes, *n* (%)	10 (21.3)
Vascular disease, *n* (%)	1 (2.1)
Stroke, *n* (%)	4 (8.5)
Heart failure, *n* (%)	11 (23.4)
Structural heart disease, *n* (%)	6 (12.8)
Hypertrophic cardiomyopathy, *n* (%)	4 (8.5)
Valvular heart disease, *n* (%)	2 (4.3)
CHADS_2_ score	1.6 ± 1.3
CHA_2_DS_2_‐VASc score	3.0 ± 1.7
**Echocardiography findings**	
Left ventricular ejection fraction (%)	63.2 ± 7.1
Left atrial diameter (mm)	43.8 ± 5.4
**Medication before procedure**	
Antiarrhythmic drugs	
Class 1 antiarrhythmic drugs, *n* (%)	1 (2.1)
Amiodarone, *n* (%)	3 (6.4)
Beta blocker, *n* (%)	21 (44.7)
Others, *n* (%)	1 (2.1)
ACE inhibitor or ARB, *n* (%)	27 (57.4)
Statin, *n* (%)	16 (34.0)

Abbreviations: ACE, angiotensin‐converting enzyme; AF, atrial fibrillation; ARB, angiotensin II receptor blocker.

### 3.2 Overall Bi‐Atrial Distribution of Arrhythmogenic Substrate

**FIGURE 3:**
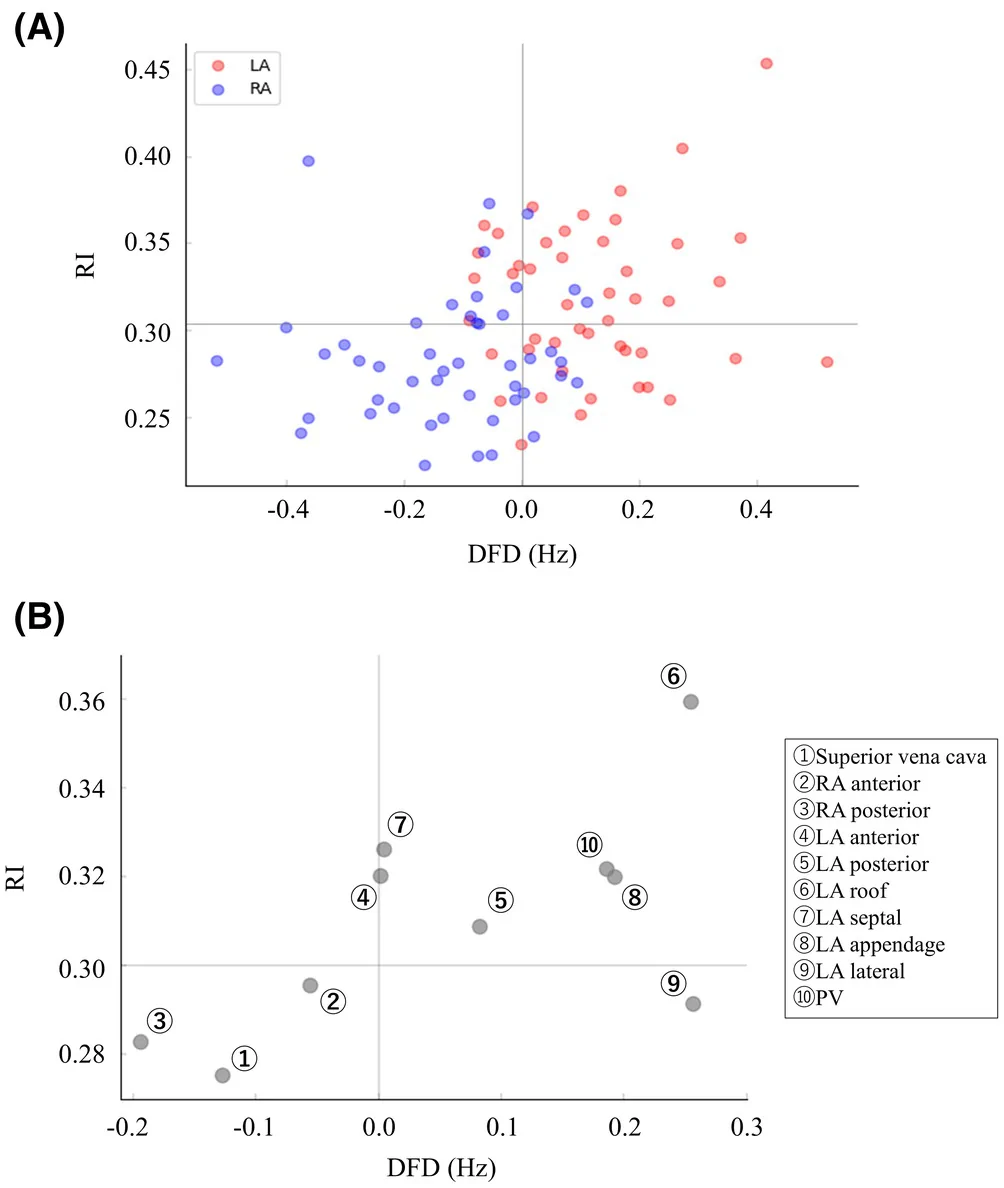
Scatter plot of spectral characteristics in both atria during AF. (A) Patient‐level mean DFD and RI for the LA (red) and RA (blue). (B) Mean DFD and RI for each of the 10 anatomical segments. The LA roof showed the highest DFD and RI among all segments. AF, atrial fibrillation; DFD, dominant frequency deviation; LA, left atrium; PV, pulmonary vein; RA, right atrium; RI, regularity index.

CARTOFINDER evaluated a total of 48 153 mapping points across both atria. Comparison of spectral characteristics between the RA and LA revealed that the LA exhibited significantly higher DFD (0.12 ± 0.69 Hz vs. −0.13 ± 0.73 Hz, *p* < 0.001) and RI (0.32 ± 0.11 vs. 0.29 ± 0.10, *p* < 0.001) than the RA (Figure 3A). Among all 10 segments, the LA roof demonstrated the highest DFD and RI, followed by the LA appendage, PV, and LA posterior wall (Figure 3B, Table 2). Notably, the mean DFD of the LA septum was 0.00 ± 0.56 Hz, indicating that this site most closely approximates the subject‐level mean DF, functioning as an atrial “reference point.” These findings suggest that the LA, particularly the LA roof, harbors the most critical electrophysiological substrate for non‐paroxysmal AF maintenance.

**TABLE 2:** Spectral gradient in both atria during AF.

	DFD (Hz)	RI
Overall	0.00 ± 0.00	0.30 ± 0.11
Superior vena cava	−0.13 ± 0.72	0.28 ± 0.10
RA anterior	−0.06 ± 0.75	0.30 ± 0.10
RA posterior	−0.19 ± 0.71	0.28 ± 0.10
LA anterior	0.00 ± 0.61	0.32 ± 0.12
LA posterior	0.08 ± 0.68	0.31 ± 0.10
LA roof	0.25 ± 0.61	0.36 ± 0.12
LA septal	0.00 ± 0.56	0.33 ± 0.10
LA appendage	0.19 ± 0.75	0.32 ± 0.13
LA lateral	0.26 ± 0.85	0.29 ± 0.11
PV	0.19 ± 0.70	0.32 ± 0.11

Abbreviations: AF, atrial fibrillation; DFD, dominant frequency deviation; LA, left atrium; PV, pulmonary vein; RA, right atrium; RI, regulatory index.

### 3.3 Distribution and Spectral Characteristics of FoA

**FIGURE 4:**
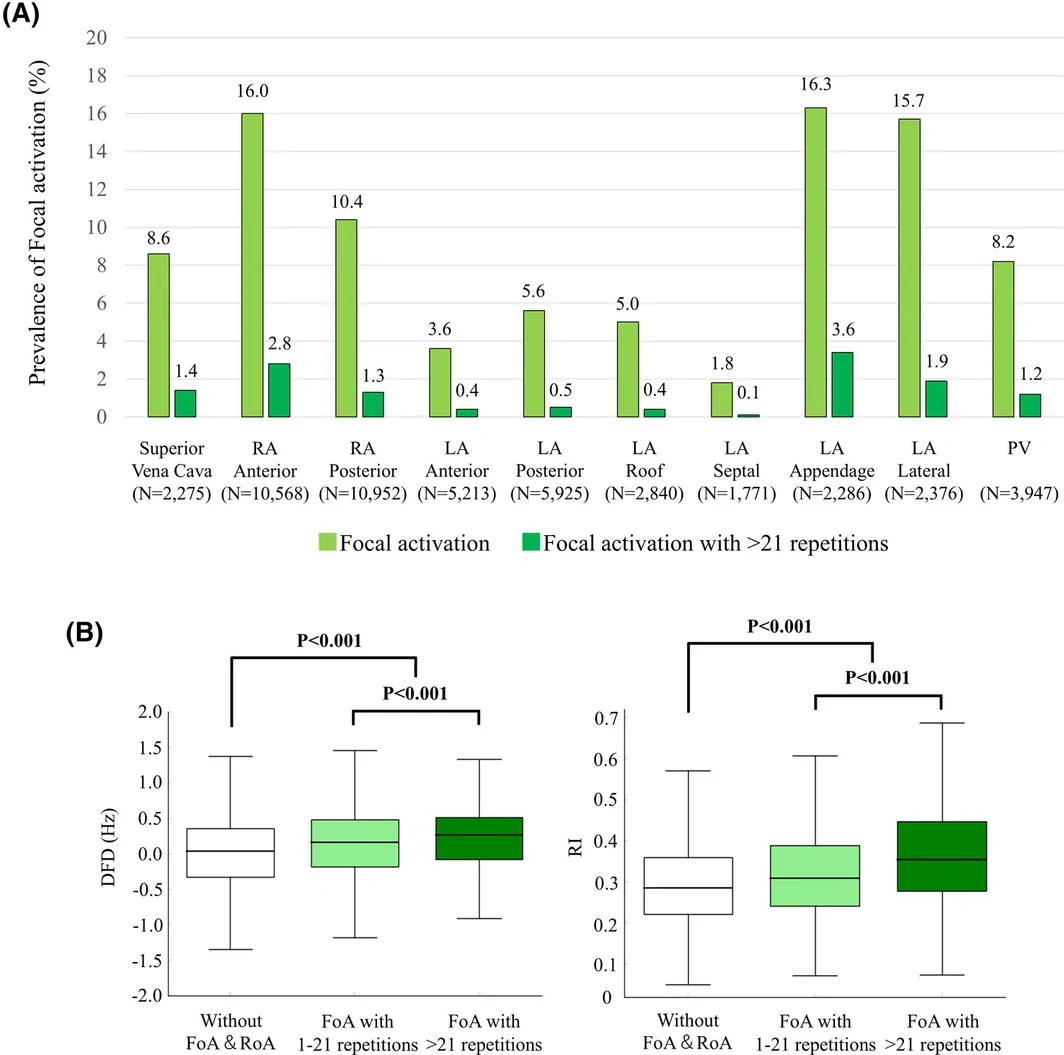
Distribution and spectral characteristics of FoA. (A) Prevalence of FoA (light green) and high‐repetition FoA (> 21 repetitions; dark green) in each segment. (B) Box‐and‐whisker plots of DFD and RI stratified by FoA repetition count. Both DFD and RI increased stepwise with increasing FoA repetitions (both *p* < 0.001). DFD, dominant frequency deviation; FoA, focal activation; LA, left atrium; PV, pulmonary vein; RA, right atrium; RI, regularity index; RoA, rotational activation.

FoA was identified in all 47 patients (100%), predominantly distributed in the LA appendage, RA anterior wall, and LA lateral wall (Figure 4A). Overall, FoA sites exhibited significantly higher DFD and RI than sites without FoA and RoA (both *p* < 0.001). Subgroup analysis stratified by repetition count revealed that DFD and RI increased in a stepwise and significant manner as FoA repetitions increased (both *p* < 0.001). In particular, high‐repetition FoA sites (> 21 repetitions) demonstrated the highest DFD and RI compared with low‐repetition sites (Figure 4B, Table 3). This consistent pattern—in which greater FoA repetition is associated with faster and more regular activation—supports the interpretation that high‐repetition FoA sites represent active AF drivers.

**TABLE 3:** Spectral characteristics of FoA and RoA sites.

	DFD (Hz)	RI
Sites without FoA&RoA	−0.03 ± 0.72	0.30 ± 0.10
FoA sites	0.16 ± 0.67	0.33 ± 0.11
with 1–21 repetitions	0.14 ± 0.68	0.32 ± 0.11
with > 21 repetitions	0.26 ± 0.56	0.37 ± 0.12
RoA sites	0.25 ± 0.73	0.37 ± 0.12
with 1–16 repetitions	0.26 ± 0.74	0.36 ± 0.12
with > 16 repetitions	0.14 ± 0.64	0.42 ± 0.13

Abbreviations: AF, atrial fibrillation; DFD, dominant frequency deviation; FoA, focal activation; RI, regulatory index; RoA, rotational activation.

### 3.4 Distribution and Paradoxical Spectral Changes of RoA

**FIGURE 5:**
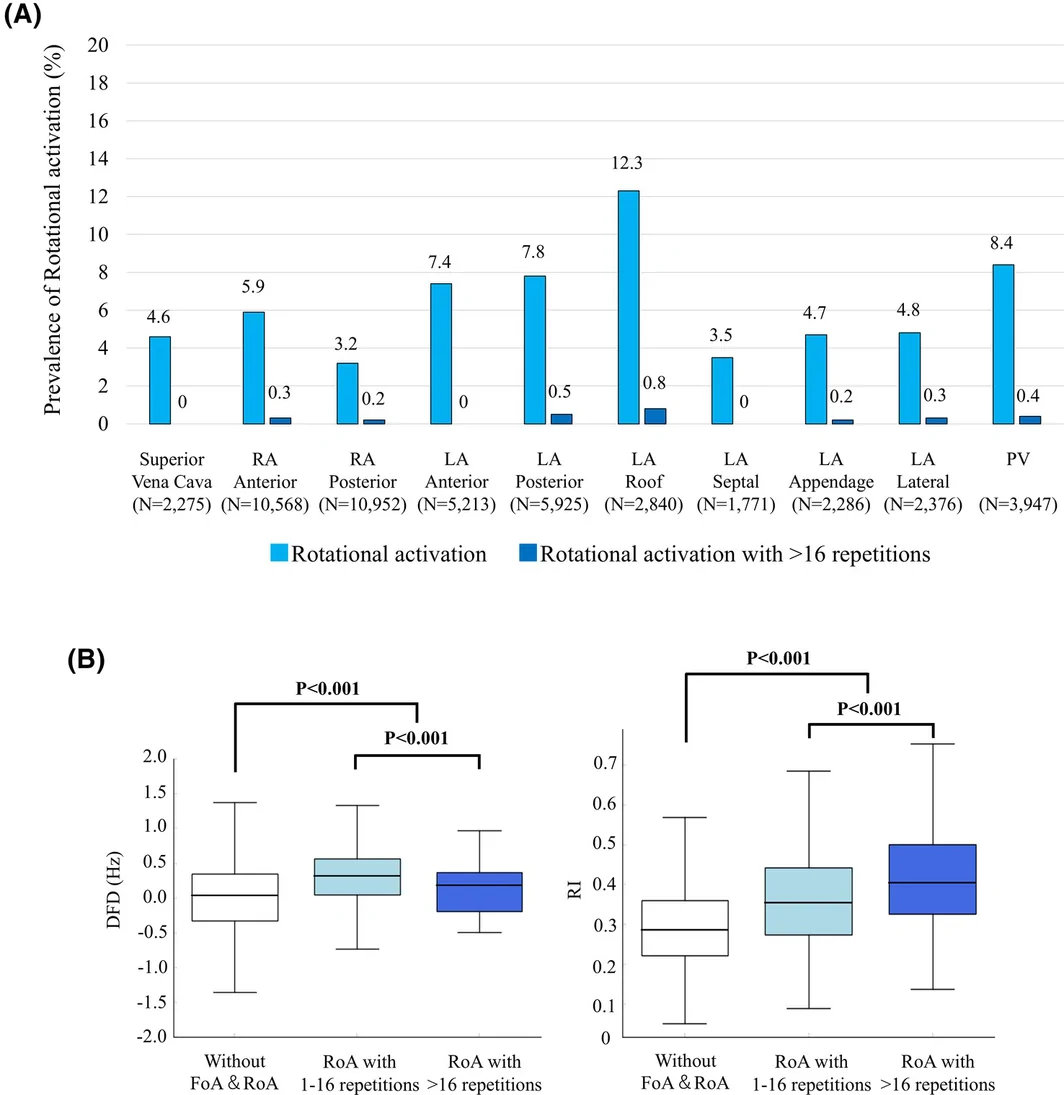
Distribution and spectral characteristics of RoA. (A) Prevalence of RoA (light blue) and high‐repetition RoA (> 16 repetitions; dark blue) in each segment. (B) Box‐and‐whisker plots of DFD and RI stratified by RoA repetition count. High‐repetition RoA sites showed a paradoxical decrease in DFD despite a significant increase in RI compared with low‐repetition RoA sites (both *p* < 0.001). DFD, dominant frequency deviation; FoA, focal activation; LA, left atrium; PV, pulmonary vein; RA, right atrium; RI, regularity index; RoA, rotational activation.

RoA was identified in 25 of 47 patients (53.2%), predominantly distributed in the LA roof, PV, and LA posterior wall (Figure 5A). Overall, RoA sites exhibited significantly higher DFD and RI than sites without FoA and RoA (both *p* < 0.001). However, repetition‐stratified analysis revealed a paradoxical pattern contrasting with that of FoA. High‐repetition RoA sites (> 16 repetitions) demonstrated a significantly higher RI than low‐repetition RoA sites (0.42 ± 0.13 vs. 0.36 ± 0.12, *p* < 0.001), yet paradoxically exhibited a significantly lower DFD (0.14 ± 0.64 Hz vs. 0.26 ± 0.74 Hz, *p* < 0.001) (Figure 5B, Table 3). Thus, high‐repetition RoA sites exhibit spectral characteristics fundamentally distinct from those of FoA: They are more organized yet relatively slower in activation rate.

## 4 Discussion

### 4.1 Principal Findings

To our knowledge, this is the first prospective study to integrate high‐density CARTOFINDER mapping of both atria using the Octaray catheter with offline spectral analysis. The principal findings are threefold: (i) the LA demonstrated significantly higher DFD and RI than the RA, with the LA roof identified as the most critical arrhythmogenic substrate region; (ii) DFD and RI increased progressively with increasing FoA repetitions, indicating that high‐repetition FoA sites function as active AF drivers; and (iii) high‐repetition RoA sites exhibited paradoxically lower DFD despite higher RI, reflecting a mechanistic origin fundamentally distinct from that of FoA.

### 4.2 Left Atrial Predominance of Spectral Gradient and Significance of the LA Roof

The finding that the LA exhibited higher DFD and RI than the RA is consistent with multiple prior reports [12, 13]. The LA has a complex anatomical architecture, and the LA roof in particular, situated at the junction of the pulmonary veins and the posterior atrial wall, is prone to wavefront collision and fusion from multiple directions, providing an anatomical substrate for rotor formation [14]. Indeed, in the present study, although FoA was not predominantly distributed at the LA roof, this region was the principal location of RoA and recorded the highest DFD and RI of all segments, suggesting that the LA roof functions as a central “hub” of reentrant circuitry.

### 4.3 Interpretation of FoA: Active Electrical Discharge From Intramural Rotors

The pattern in which both DFD and RI increased with greater FoA repetition suggests that high‐repetition FoA sites do not merely represent passive wavefront collisions, but rather constitute true drivers that actively discharge electrical impulses from intramural rotors toward the endocardial surface. The observation that high‐frequency FoA activates faster than the surrounding myocardium while maintaining high regularity is consistent with the electrophysiological behavior expected of a dominant local source [15]. Accordingly, high‐repetition FoA sites should be proactively identified and targeted as priority ablation sites.

### 4.4 Reentrant Circuits Associated With Structural Remodeling: A Mechanistically Distinct Substrate

The paradoxical finding of decreasing DFD with increasing RoA repetition suggests that high‐repetition RoA sites maintain their reentrant circuits on a scaffold of low‐voltage zones and scar tissue formed by atrial structural remodeling and fibrosis, rather than through active driver activity [16]. Whether these circuits play an active stabilizing role in AF maintenance cannot be definitively excluded from the present data. The observed pattern—lower DFD combined with higher RI—is consistent with conduction slowing imposed by fibrotic tissue, reflecting a mechanism fundamentally distinct from that of FoA [17]. High‐repetition RoA sites may serve as markers of advanced atrial remodeling and represent an important, if mechanistically distinct, therapeutic target.

### 4.5 Mechanistic Basis for Potential Ablation Target Identification

CARTOFINDER identifies focal and rotational activation patterns based on wavefront morphology and repetition, but does not quantify local activation rate or conduction homogeneity. Spectral analysis provides complementary characterization: Among high‐repetition FoA sites, elevated DFD may suggest active driver activity driven by rapid focal discharge, whereas high‐repetition RoA sites with paradoxically low DFD and high RI suggest reentrant circuits governed by conduction slowing within fibrotic substrate. These spectral profiles thus allow mechanistic differentiation of substrate types that repetition count alone cannot resolve.

As supplementary data, we observed that AF termination during ablation occurred in 12.8% (6/47) of patients, exclusively at high‐repetition FoA sites (Table S1). However, several randomized trials evaluating CARTOFINDER‐guided ablation, including the MAP‐AF trial [7], have demonstrated neutral outcomes compared with conventional strategies, highlighting the difficulty of translating mechanistic substrate mapping into clinical benefit. As Lin YJ et al. demonstrated [18], diffuse substrate remodeling in non‐paroxysmal AF may reduce the spatial stability of DF gradients, potentially limiting the reliability of DF‐based localization in this population. This framework remains hypothesis‐generating, and prospective validation is required before spectral characterization can be integrated into real‐time ablation planning.

### 4.6 Application to Intraoperative Real‐Time Assessment: DF And RI Approximation Formulas

Performing complex offline spectral analysis in real time during the procedure is currently impractical. However, the present study demonstrated that the AF cycle length (CL) and its standard deviation (CLSD)—both of which can be readily measured on the CARTO system—exhibit significant linear correlations with DF and RI, respectively (Figure S3).

$$\mathrm{DF}\;\left(\mathrm{Hz}\right)≒-0.032\times \mathrm{CL}\;\left(\mathrm{ms}\right)+11.25$$

$$\mathrm{RI}≒-0.034\times \text{CLSD}\;\left(\mathrm{ms}\right)+1.33$$

Using these approximation formulas, the operator can estimate DF and RI at each site in real time from CL and CLSD displayed on the CARTO screen, without the need for time‐consuming offline analysis, thereby enabling intraoperative prioritization of ablation targets. Furthermore, the observation that the mean DFD of the LA septum was 0.00 ± 0.56 Hz suggests that the DF measured at the LA septum may serve as a surrogate for the subject‐level mean DF (reference value) for that patient. This would allow the operator to assess the relative DFD (an index of substrate activity) at any given site simply by measuring the DF at the LA septum during the procedure, facilitating further intraoperative simplification. However, these approximation formulas have not been prospectively validated in independent datasets. Their potential clinical utility is therefore exploratory and hypothesis‐generating.

### 4.7 Limitations

Several limitations of this study warrant acknowledgment. First, the relatively small sample size (*n* = 47) limits the generalizability of our findings. Although the total number of mapping points is large (48 153), the effective unit of analysis remains at the patient level, and subgroup comparisons stratified by repetition count should be interpreted with caution. These findings should be regarded as hypothesis‐generating and require confirmation in larger cohorts. Second, because CARTOFINDER mapping was performed sequentially across atrial regions (mean total mapping time 27.0 ± 6.1 min; RA: 12.8 ± 3.4 min; LA: 14.1 ± 3.9 min; Table S2), AF wavefront dynamics may have changed during the course of mapping. Given the non‐stationary nature of AF, activation patterns—including detection and repetition counts of FoA and RoA—may vary over time, potentially underestimating true driver prevalence. Third, while DFD was introduced to account for the substantial inter‐subject variability in mean DF observed in our cohort, we acknowledge that this normalization may obscure clinically meaningful absolute differences in activation rate between patients. Sub‐analyses using absolute DF values yielded directionally consistent findings, supporting DFD as a valid relative index of local substrate activity (Figure S4). Fourth, residual pharmacological effects of amiodarone in some patients cannot be entirely excluded, which may have influenced spectral characteristics. Fifth, post‐ablation remapping was not performed, and we therefore cannot assess whether identified substrate sites were eliminated or altered following ablation. Finally, this study is mechanistic and exploratory in nature. Although supplementary outcome data are provided for reference, the offline spectral analysis was performed post hoc and did not guide ablation decisions. Whether integrating spectral characterization into real‐time ablation planning improves clinical outcomes requires validation in prospective randomized trials.

## 5 Conclusions

FoA and RoA sites identified by CARTOFINDER using the Octaray catheter exhibited significantly higher DFD and RI compared with non‐substrate sites. DFD and RI increased progressively with greater FoA repetition, consistent with the role of high‐repetition FoA sites as active AF drivers. Conversely, high‐repetition RoA sites displayed a paradoxical decrease in DFD despite elevated RI, suggesting a mechanism associated with structural atrial remodeling; whether these sites actively contribute to AF maintenance remains to be determined. The LA roof was identified as the most critical substrate region across both atria. The combination of CARTOFINDER mapping and spectral analysis may contribute to the mechanistic understanding of non‐paroxysmal AF.

## Funding

This work was supported by Johnson and Johnson.

## Disclosure

This study was approved by the institutional ethics committee of Kyoto Prefectural University of Medicine (research project no. ERB‐C‐2609). Written informed consent was obtained from all patients.

## Conflicts of Interest

This work was supported by Johnson & Johnson MedTech Research Grant. All authors have reported that they have no relationships relevant to the contents of this paper to disclose.

## Supporting information


**Table S1:** Details of patients with AF termination during the procedure.
**Table S2:** Details of the CARTOFINDER mapping data.

**Figure S1:** Mean DF between subjects. DF, dominant frequency.

**Figure S2:** Concept of DFD. Substantial inter‐subject variability existed in mean DF. Therefore, the subject‐level mean DF (mean DF1 and mean DF2) was designated as the reference (zero baseline) for those subjects, and the deviation of each mapping point's DF from this reference was defined as DFD (DFD1 and DFD2). DF, dominant frequency; DFD, dominant frequency deviation.

**Figure S3:** Correlation between the CL domains and spectral domains. (A) Correlation between the CL and DF. (B) Correlation between the CLSD and RI. CL, cycle length; CLSD, cycle length standard deviation; DF, dominant frequency; RI, regularity index.

**Figure S4:** Box‐and‐whisker plots of DF stratified by FoA and RoA repetition count. DF significantly increased stepwise with increasing FoA repetitions. High‐repetition RoA sites showed a paradoxical decrease in DF compared with low‐repetition RoA sites (*p* < 0.001). DF, dominant frequency; FoA, focal activation; RoA, rotational activation.

## Data Availability

The deidentified participant data will not be shared.
