# Supplementary material for: High‐Density Spectral Analysis for Bi‐Atrial Arrhythmogenic Substrate During Non‐Paroxysmal Atrial Fibrillation
Source: J Arrhythm. 2026 Sep 21;42(5):e70477. doi: 10.1002/joa3.70477 (PMC13591222; doi:10.1002/joa3.70477)
Supplement: Supplementary file 1 [file JOA3-42-e70477-s001.docx]

**Table S1. Details of patients with AF termination during procedure**

| No. | Age, y | Sex | Diagnosis | Types of  AF termination | Types of  AF driver | Location with  AF termination | Additional procedure |
| --- | --- | --- | --- | --- | --- | --- | --- |
|  |  |  |  |  |  |  |  |
| 1 | 54 | M | PeAF | Sinus | HR-FoA | On the PVI line | - |
| 2 | 68 | F | LPeAF | Focal AT | HR-FoA | LA posterior | Point ablation |
| 3 | 74 | F | PeAF | RA scar-related AT | HR-FoA | LA lateral | Linear ablation |
| 4 | 72 | M | LPeAF | Sinus | HR-FoA | On the PVI line | - |
| 5 | 51 | M | LPeAF | Roof-dependent AT | HR-FoA | LAA ostium | LA posterior wall isolation |
| 6 | 74 | F | PeAF | Focal AT | HR-FoA | RA posterior | Point ablation |

AF=atrial fibrillation, AT=atrial tachycardia, F=female, HR-FoA=high-repetitive focal activation, LA=left atrium, LAA= left atrium appendage, LPeAF=long-standing persistent atrial fibrillation, M=male, PeAF=persistent atrial fibrillation, PVI=pulmonary vein isolation, RA=right atrium

**Table S2. Details of the CARTOFINDER mapping data**

|  | Number of maps (/patient) | Mapping time (min) |
| --- | --- | --- |
| Overall | 31.2 ± 5.6 | 27.0 ± 6.1 |
| RA | 14.4 ± 2.8 | 12.8 ± 3.4 |
| Superior vena cava | 1.8 ± 1.1 | - |
| RA anterior | 5.9 ± 1.9 | - |
| RA posterior | 6.6 ± 1.7 | - |
| LA | 16.9 ± 3.4 | 14.1 ± 3.9 |
| LA anterior | 3.8 ± 1.3 | - |
| LA posterior | 3.8 ± 1.2 | - |
| LA roof | 1.9 ± 0.9 | - |
| LA septal | 1.3 ± 0.6 | - |
| LA appendage | 1.3 ± 0.5 | - |
| LA lateral | 1.5 ± 0.6 | - |
| PV | 3.2 ± 1.0 | - |

LA=left atrium, PV=pulmonary vein, RA=right atrium
